# Placebo design in WHO-registered trials of Chinese herbal medicine need improvements

**DOI:** 10.1186/s12906-019-2722-2

**Published:** 2019-11-06

**Authors:** Xuan Zhang, Ran Tian, Chen Zhao, Xudong Tang, Aiping Lu, Zhaoxiang Bian

**Affiliations:** 1 Chinese Clinical Trial Registry (Hong Kong), Hong Kong Chinese Medicine Clinical Study Centre, School of Chinese Medicine, Hong Kong Baptist University, Jockey Club School of Chinese Medicine Building, 7 Baptist University Road, Kowloon Tong, Kowloon Hong Kong, China; 2 Xiyuan Hospital, China Academy of Chinese Medical Sciences, Beijing, 100091 China

**Keywords:** Placebo, Chinese herbal medicine (CHM), Traditional Chinese medicine (TCM), Clinical trial registration, WHO ICTRP

## Abstract

**Background:**

Physical identical and pharmacological inert are the basic requirements for placebo design, which are essential in clinical trials to evaluate the efficacy of an intervention. However, it is difficult to makeup a placebo of Chinese herbal medicine (CHM) because of special color, taste and smell, etc. Currently, there is no specific requirements and standards for the creation of a CHM-placebo. The purpose of this study is to review the characteristics of the CHM-placebo design and application in registered clinical trials with CHM interventions and identify the common problems, if any.

**Methods:**

The World Health Organization (WHO) International Clinical Trials Registry Platform (ICTRP) was systematically searched for CHM interventional trials with placebo-controlled design up to 31 December 2017. Registered information of each included trial was collected from specific registries involved in ICTRP through hyperlinks. Descriptive statistics were used to analyze the characteristics of placebo design in CHM trial registrations.

**Results:**

A total of 889 CHM interventional trials were registered from 1999 to 2017, and 40.8% (363) of them included CHM-placebo control design. The common ways of their design were: placebo as sole control (191, 52.6%); placebo as add-on control with baseline treatment (84, 23.1%); and placebo as double-dummy control (57, 15.7%). Among 363 included trials, 46 (12.7%) reported the compositions of placebos, including CHM ingredients (17 trials), excipients and other agents (29 trials). 2 (0.6%) reported pharmacological inert testing, and 52 (14.3%) descripted their placebos to be physically identical with the CHMs. 14 (3.9%) reported quality control of placebos, and 2 (0.6%) provided blinding assessment of placebos.

**Conclusions:**

The placebos included in most CHM trial registrations is not optimal in terms of placebo design, application, evaluation and reporting. Specific guidelines or standards of CHM-placebo design, including usage requirements, preparation specifications, quality assessments and reporting guidelines should be developed thus to improve their quality.

## Background

Although Chinese herbal medicine (CHM) is increasingly popular in the world, its effectiveness continues to be debated. The evidence to support CHM treatment approaches must come from high quality randomized, double-blinded, and placebo-controlled clinical trials [1]. Criticism of the quality of placebos used is unfortunately common. Poor design of the placebo affects the success of blinding and the efficacy of interventions adopted in the trial [2]. In addition, for many CHM clinical trials, creating a quality placebo is extraordinarily difficult because the herbs often have special colors, tastes and smells [3].

The word “*placebo*” originates from Latin; it means “*I shall please*”. It was first used in the 14th century, but did not appear in a documented medical record until the late 18th century [4]. Today, the term “*placebo*” refers to a harmless pill, medicine, or procedure with no therapeutic effect; it is used in two contexts: (a) prescribed to patients for psychological benefit; or (b) used in clinical trials as a way to test the efficacy of new drugs [5].

In 1970, the American Food and Drug Administration (FDA) suggested that under the consideration of ethical approval, placebo control design should be used in clinical studies for new drug evaluation [6]. In 2000, the fifth revision of the *Declaration of Helsinki* addressed the appropriate use of placebos [7]. It requires the application of placebo in clinical studies when there is no established effective intervention; when withholding effective intervention would mostly expose subjects to temporary discomfort or delay in relief of symptoms; when use of effective intervention would not yield scientifically reliable results; and when the use of placebo would not add any serious or irreversible harm to the subjects [8]. For randomized controlled trials (RCTs), a placebo group is designed to control for several factors, including placebo effects, statistical regression to the mean, spontaneous remission, etc. [9, 10]. Thus, if a clinical trial is supported by sound ethical and methodological considerations, a placebo control design is the most rigorous test of treatment efficacy, especially for evaluating a medicinal therapy [11].

In traditional Chinese medicine (TCM), the first CHM intervention clinical trial with a CHM-placebo control design was published in 1985; it examined the use of Suan-Zao-Ren-Tang in treating insomnia [12]. In 1999, the China Food and Drug Administration (CFDA) issued a document of “Technical Requirements for Clinical Research of New Drugs in Traditional Chinese Medicine”, which encouraged placebo design, as a comparator product, in the phase II of clinical trials, if necessary [13]. Since then, an increasing number of CHM interventional trials with placebo control have been designed and implemented [14]. The first registration of a CHM trial with placebo control was in 2002; the trial was conducted to test the efficacy of a CHM formula (a capsule included 11 herbs) against Crohn’s disease [15].

A good placebo would be identical to the real CHM intervention in physical form, sensory characteristics, packaging, and labeling, and it would have no pharmaceutical activity [16]. However, few studies have reported these information of a placebo [17]. For example, Wu SP et al. have analyzed 301 CHM placebo-controlled clinical trials published from 1983 to 2013, and have found that only 3 articles (1.0%) reported the testing information on pharmacologically inert and physical similarity of CHM-placebos [18]. A similar study conducted by Qi GD et al., examining 77 CHM placebo-controlled clinical trials from 1999 to 2005, found quite similar results [19]. Besides, some studies have assessed the ethical requirements of placebo application in CHM clinical trials and have found some undesirable results [20]. For instance, Fu JJ et al. have reported that, among 231 CHM placebo-controlled trials published in Chinese journals from 1979 to 2008, 48 (20.78%) did not meet the scientific requirement of placebo usage, 221 (95.67%) did not report ethic approval, and 187 (80.95%) did not report information on informed consent in the publications [21]. Based on literature review, we found that there is no latest study so far to provide the current characteristics of placebo design and its usage in CHM trials, especially in registered CHM clinical trials.

This study aimed to review the overall characteristics of placebo design and application in CHM clinical trial registrations. The objectives were as follows: 1) to summarize the general features of placebo design and application in WHO-registered CHM trials; 2) to identify the common problems of placebo design and application in WHO-registered CHM trials; and 3) to provide suggestions for improving the quality of CHM-placebo design and their use in the future. These results will be the basis for setting up specific guidelines or standards for the CHM-placebo design.

## Methods

### Study design and setting

The WHO ICTRP was searched for registered CHM clinical trials with placebo-controlled design up to 31 December 2017 in this study, and descriptive statistics were used to analyze their characteristics of placebo design.

### Data source

The database of the WHO ICTRP (http://apps.who.int/trialsearch/) was searched on 15 January 2018 for all TCM trials that had been registered up to 31 December 2017. There are 17 Registries in the ICTRP: Australian New Zealand Clinical Trials Registry (ANZCTR), Chinese Clinical Trial Register (ChiCTR), ClinicalTrials.gov, EU Clinical Trials Register (EU-CTR), International Standard Randomized Controlled Trial Number Register (ISRCTN), the Netherlands National Trial Register (NTR), Brazilian Clinical Trials Registry (ReBec), Clinical Trials Registry-India (CTRI), Clinical Research Information Service-Republic of Korea (CRiS), Cuban Public Registry of Clinical Trials (RPCEC), German Clinical Trials Register (DRKS), Iranian Registry of Clinical Trials (IRCT), Japan Primary Registries Network (JPRN), Pan African Clinical Trial Registry (PACTR), Sri Lanka Clinical Trials Registry (SLCTR), Thai Clinical Trials Register (TCTR), Peruvian Clinical Trials Registry (REPEC).

### Ethical considerations

This study was designed to analyze the overall characteristics of placebo design in CHM clinical trial registrations from the public access way of the ICTRP. All data used in this study are registered information related to trial design and does not involve human subjects.

### Search strategy

Standard search, provided by WHO ICTRP (ICTRP Search Portal, http://apps.who.int/trialsearch/) was selected and the search strategy was developed including ‘Chinese medicine OR traditional Chinese medicine OR Chinese materia medica OR Chinese herbal medicine OR acupuncture OR moxibustion OR tuina OR massage OR cupping OR guasha’, without any restrictions.

### Inclusion and exclusion criteria

We searched all TCM clinical trials registered up to 31 December 2017 and identified CHM interventional studies according to the ‘study type’ (e.g. interventional, observational, etc.) and ‘intervention’ (e.g. Chinese herbal medicine, acupuncture, cupping, etc.). The CHM interventions included Chinese medicinal substances (e.g. single herbs or extracts from single herbs) and CHM compound formulas (e.g. fixed, individualized or patent proprietary formulas). The dosage forms of CHM interventions included decoctions, capsules, pills, powders, granules, ointments, and injections, etc. However, the CHM interventional trials that included CHM-placebo control design were eligible for inclusion. There were no limitations in the participants and outcomes. We excluded the following registered TCM trials: non-interventional studies (e.g. observational studies); studies with non-CHM interventions, such as acupuncture, moxibustion, massage, cupping, etc.; and CHM interventional studies without placebo of CHM (e.g. CHM vs active control, CHM vs conventional drug vs placebo of conventional drug).

### Data extraction and analyses

Using a predefined data extraction form that collected information for this study, two authors (XZ and RT) extracted the data from each trial record independently. Disagreements were resolved by consensus. If needed, a third author (CZ) was consulted. The form of data extraction was composed of two parts: (1) Characteristics of the included trials (e.g. diseases studied, CHM interventions, control group, outcomes, study phase and ethic approval, etc.); and (2) Characteristics of the CHM placebos, including placebo compositions, pharmacological testing for inertness, physical similarity (i.e. should be identical), quality control and manufacturer of the placebo, and successful blinding assessment, etc. All data were collected and recorded in Microsoft Office Excel (Version 2016). Categorical data is presented as number (n) and percent (%).

## Results

### Search

**Fig. 1:**
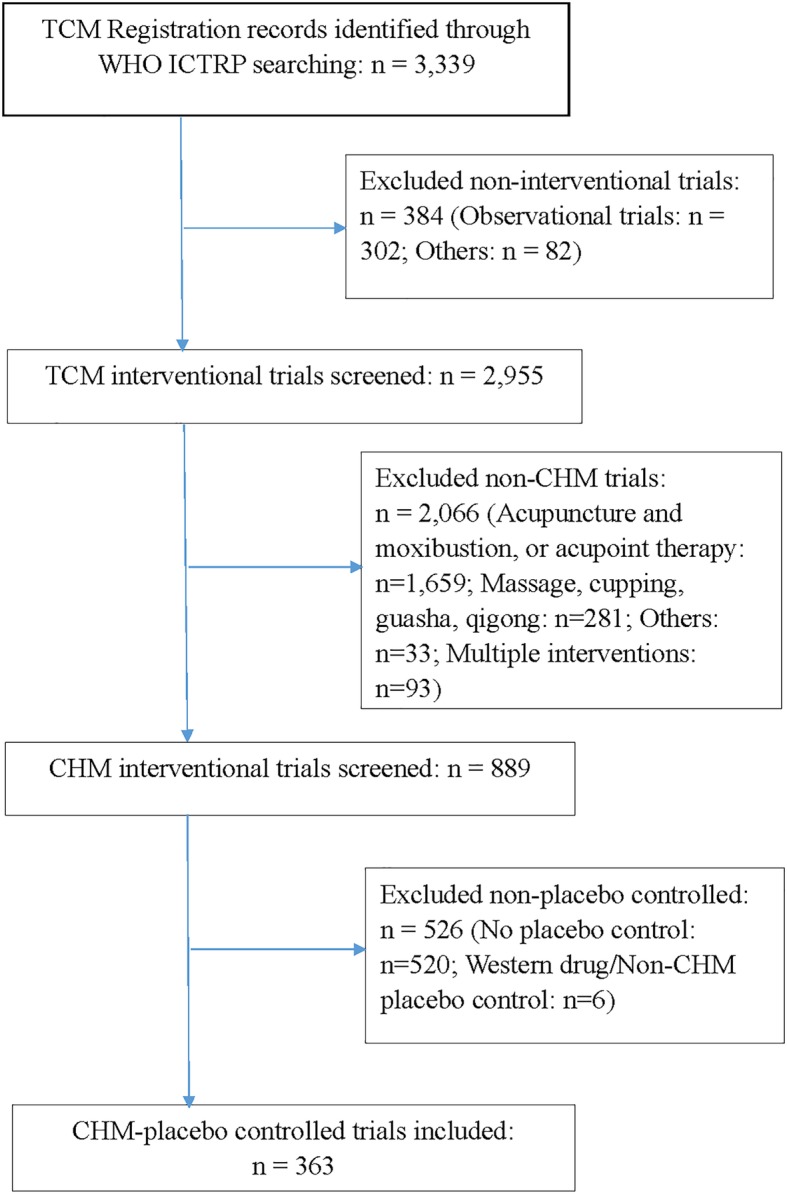
Flow chart of data identified, included and excluded

The initial search identified 3339 records. Screening excluded 384 records that were non-interventional studies. After examination of 2955 interventional studies, 889 trials with CHM interventions were chosen for further screening. A total of 363 trials (40.8%, 363/889) were included because of placebo-controlled design with CHM interventions (Fig. 1). An ID list of all included records is provided in Additional file 1: S_1_.

### Distribution of years and registries

**Fig. 2:**
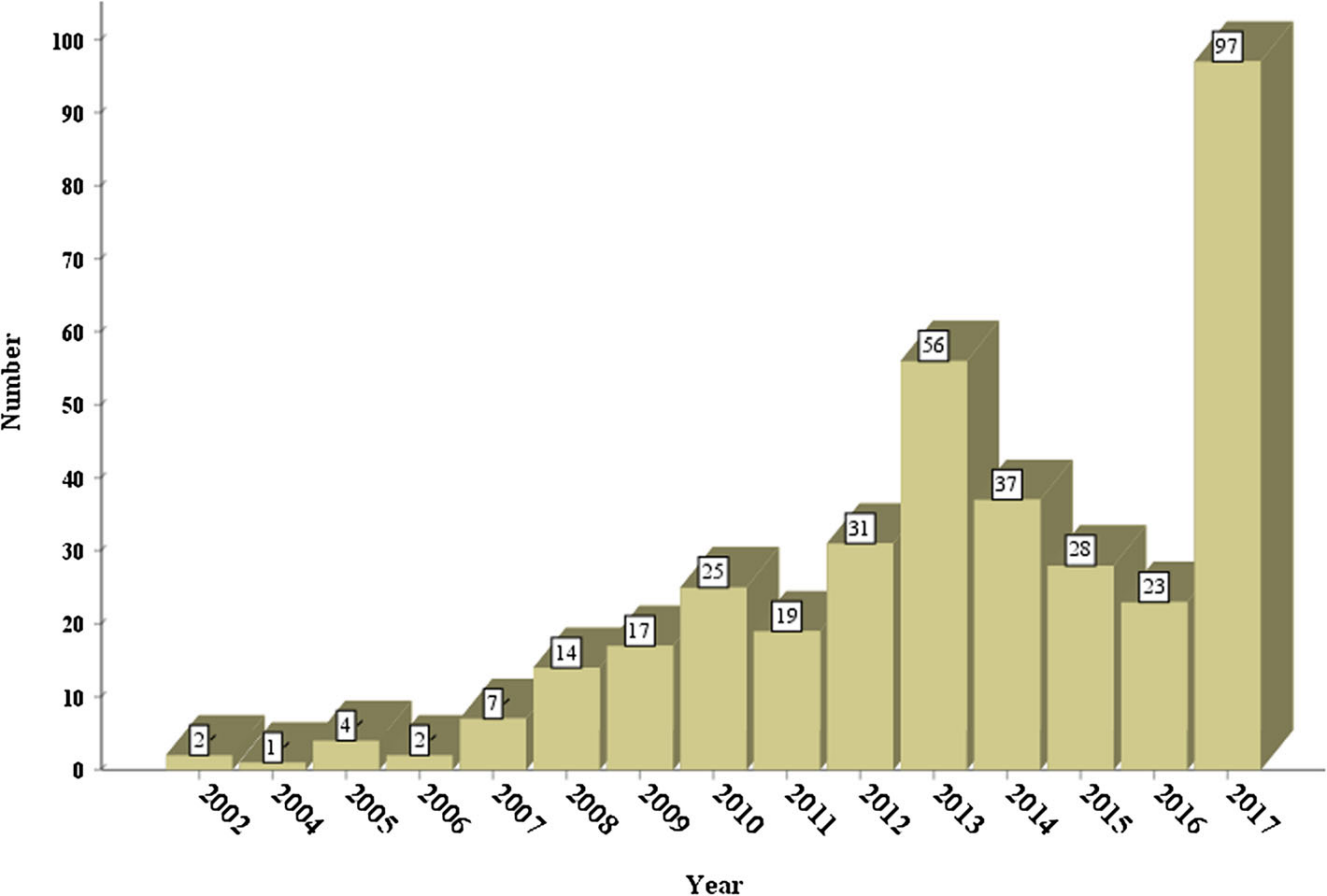
Number of registered CHM-placebo controlled clinical trials from 2002 to 2017

**Fig. 3:**
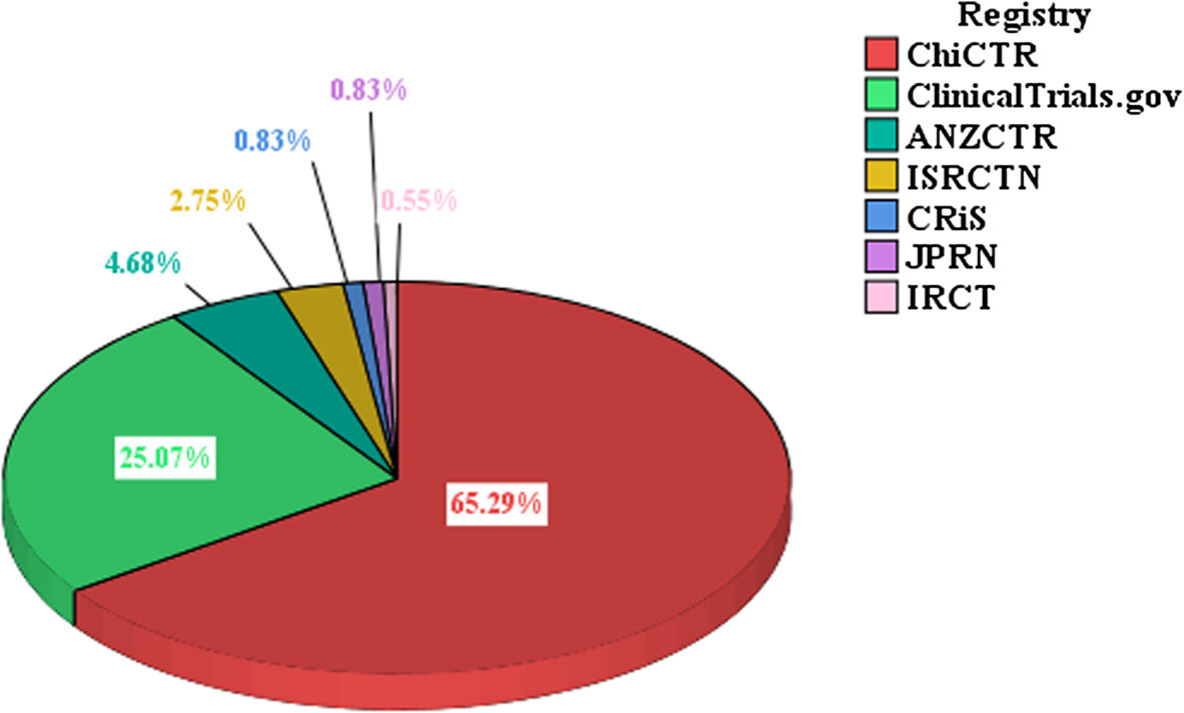
Distribution of CHM-placebo controlled trials in 7 registries from 2002 to 2017

A total of 363 CHM placebo-controlled trials were registered during the period of 2002 to 2017. The number of these trial registrations increased gradually during the first 10 years, and then increased rapidly from 2013 to 2017 (66.4%, 241/363), especially in 2013 (15.4%, 56/363) and 2017 (26.7%, 97/363) (Fig. 2). Among 17 WHO registries, CHM placebo-controlled trial registrations were only found in 7 registries, namely ChiCTR (237), ClinicalTrials.gov (91), ANZCTR (17), ISRCTN (10), CRiS (3), JPRN (3) and IRCT (2). The number of ChiCTR (i.e. China) and ClinicalTrials.gov (i.e. USA) altogether accounted for 90.4% (328/363) of all CHM placebo-controlled trial registrations (Fig. 3).

### General characteristics of CHM placebo-controlled trials

The CHM interventions were classified as either CHM formulas (88.4%, 321/363) or single herbs (11.6%, 42/363). Most (95.9%, 348/363) of trials chose the oral route, and the common dosage forms were granule (37.5%, 136/363) and capsule (13.5%, 49/363). The most common conditions studied were the circulatory system diseases (12.4%, 45/363), followed by the digestive system diseases (9.9%, 36/363). Only 29.8% (108/363) of the included trials, however, had adopted TCM syndrome as diagnostic criteria.

Of 363 included trials, 251 (69.1%) provided their ethic approvals to the registration system; 94 (25.9%) reported study phases were II or III. The most common design was solely placebo as control (52.6%, 191/363), followed by placebo as add-on control with baseline treatment (23.1%, 84/363). For placebo-controlled groups, the sample size and administration time were mainly distributed between 1 and 100 (65.8%, 239/363) and half month-3 months (22.9%, 83/363), respectively. For different categories of outcomes, the largest proportion was distributed in both objective and subjective outcomes (63.1%, 229/363). More detailed information is shown in Table 1.

**Table 1:** Characteristics of the included trials

Category	Descriptive Characteristics	*N* = 363 (%)
CHM interventions	Single herb	42 (11.6)
	CHM formula	321 (88.4)
Dosage form ^a^	Granule	136 (37.5)
	Capsule	49 (13.5)
	Decoction	41 (11.3)
	Pill	25 (6.9)
	Tablet	23 (6.3)
	Powder	15 (4.1)
	Injection	8 (2.2)
	Cataplasm	4 (1.1)
	Plaster	4 (1.1)
	Oral-liquid	3 (0.8)
	Ointment	3 (0.8)
	Mixture	1 (0.3)
	Not reported	56 (15.4)
Administration route	Oral	348 (95.9)
	External ^b^	15 (4.1)
Conditions studied (ICD-10 codes) ^c^	Diseases of the circulatory system	45 (12.4)
	Diseases of the digestive system	36 (9.9)
	Certain infectious and parasitic diseases	35 (9.6)
	Diseases of the musculoskeletal system and connective tissue	33 (9.1)
	Diseases of the respiratory system	33 (9.1)
	Diseases of the genitourinary system	30 (8.3)
	Mental and behavioural disorders	29 (8.0)
	Neoplasms	28 (7.7)
	Endocrine, nutritional and metabolic diseases	23 (6.3)
	Diseases of the nervous system	20 (5.5)
	Diseases of the skin and subcutaneous tissue	15 (4.1)
	Symptoms, signs and abnormal clinical and laboratory findings not elsewhere classified	9 (2.5)
	Diseases of the blood and blood-forming organs and certain disorders involving the immune mechanism	7 (1.9)
	Diseases of the eye and adnexa	5 (1.4)
	Factors influencing health status and contact with health services	5 (1.4)
	Certain conditions originating in the perinatal period	4 (1.1)
	Injury, poisoning and certain other consequences of external causes	3 (0.8)
	Pregnancy, childbirth and the puerperium	2 (0.6)
	Congenital malformations, deformations and chromosomal abnormalities	1 (0.3)
Specialist classification	Internal medicine	288 (79.3)
	Gynecology	30 (8.8)
	Surgery	24 (6.6)
	Pediatrics	11 (3.0)
	Orthopedics	10 (2.8)
Diagnosis included TCM syndrome ^d^	Yes	108 (29.8)
	No	255 (70.2)
Ethics approval	Yes	251 (69.1)
	No	18 (5.0)
	Not reported	94 (25.9)
Study phase ^e^	Phase 1	52 (14.3)
	Phase 2	60 (16.5)
	Phase 3	34 (9.4)
	Phase 4	55 (15.2)
	Others	162 (44.6)
Control arm ^f^	Solely placebo as control	191 (52.6)
	Add on control with baseline treatment	84 (23.1)
	Double dummy-control ^g^	57 (15.7)
	With active control arm	43 (11.8)
	With healthy control arm	6 (1.7)
Sample size of placebo group ^h^	1–100	239 (65.8)
	101–300	107 (29.5)
	301–500	12 (3.3)
	> 500	5 (1.4)
Administration time of placebo	≤ Half month	12 (3.3)
	≤ Three months	83 (22.9)
	≤ Six months	30 (8.3)
	≤ One year	14 (3.9)
	> One year	9 (2.5)
	Not reported	215 (59.2)
Outcomes	Subjective items only	10 (2.8)
	Objective items only	121 (33.3)
	Both of objective and subjective items	229 (63.1)
	Not reported	3 (0.8)
	With TCM-related outcomes ^i^	119 (32.8)
	Adverse effects reported	114 (31.4)

^a^ Some trials included more than one CHM interventions with different dosage forms; these were counted in different columns. Thus, the total number of dosage forms was above 363

^b^ This category refers to the CHM for external use or application, such as injection, plaster and ointment

^c^ According to International Statistical Classification of Diseases and Related Health Problems 10th Revision (ICD-10) Version for 2010

^d^ According to the inclusion criteria of participants, we calculated the percentage of whether TCM syndrome were added or not

^e^ Except phase I-IV, In the registry of ChiCTR, some other options were offered for the column of study phase, such as “New treatment measure clinical study”, “Other”, etc. So, these were calculated in the item of “Others” in Table 1

^f^ Some trials included more than one category of control arm (e.g., CHM vs placebo vs healthy control; CHM vs active drug vs placebo, etc.); these were counted in different columns. Thus, the total number of this column was above 363

^g^ In the category of double-dummy control, 31 (8.5%) trials included more than one kind of placebos of CHMs, and 26 (7.2%) trials included the placebo of conventional drug

^h^ Each option includes the boundary values and range values, for example, 1–100 means “1 ≤ sample size ≤100”, namely *n* = 1 trials or *n* = 100 trials are also categorized into this column

^i^ TCM-related outcomes included TCM syndrome scores, TCM symptom scale, tongue and pulse sign, etc.

### Characteristics (composition, pharmacological effect, physical identity, blinding and quality control) of CHM placebos

As shown in Table 2, 46 trials (12.7%) reported the placebo compositions. Two trials (0.6%) conducted pharmacological inert tests. Fifty-two trials (14.3%) stated that the placebo was physically identical to the experimental CHM, especially in terms of color, smell, and taste; however, no trial reported their testing methods. Information on detailed physical characteristics (e.g. appearance, packaging, etc.) is presented in Additional file 1: S_2_. Only 2 trials (0.6%) mentioned the evaluation criteria/method for successful blinding of placebo design. Fourteen trials (3.9%) provided the information on quality control of placebo, and 10 reported the name of the company that manufactured the placebo. The relevant information on manufacturers is shown in Additional file 1: S_3_.

**Table 2:** Placebo characteristics in CHM trial registrations

Item	Report, *N* = 363 (%)	Not report, *N* = 363 (%)
Composition of placebo	46 (12.7)	317 (87.3)
Pharmacological inert test	2 (0.6)	361 (99.4)
Physically identical test ^a^	52 (14.3)	311 (85.7)
Quality control of placebo ^b^	14 (3.9)	349 (96.1)
Evaluation criteria for successful blinding of placebo	2 (0.6)	361 (99.4)

^a^ No trial reported the testing methods for determining whether the placebo was physically identical to the CHM intervention. Specific physical characteristics (e.g. color, smell, packaging, etc.) were calculated, and its detailed information is presented in Additional file 1: S_2_

^b^ 14 trials mentioned the manufacturer of placebos; thus, quality control followed the specifications of the manufacturer. Among these trials, 10 reported the specific name of placebo manufacturer, while the other 4 reported that the placebo was manufactured with GMP certification. Detailed information in this category is presented in Additional file 1: S_3_

For placebo compositions, of 46 trials, 17 (37%) trials included CHM ingredients and 29 (63%) trials excluded CHM ingredients in their placebos. The placebos including CHM ingredients mainly adopted low dosages of the experimental CHM (13, 28.3%), and 9 (19.6%) reported the specific dosage percentage of CHM ingredients. Except CHM ingredients, the placebos were mainly composed of excipients (28, 60.8%), such as flour, starch, and dextrin, plus flavoring, and/or coloring agents. Only 2 (4.3%) trials provided pharmacological testing for inertness. More detailed information is shown in Table 3.

**Table 3:** Characteristics of placebo compositions in CHM trial registrations

Placebo composition	*N* = 46(%)	Examples
With CHM ingredients	17 (37.0)	Placebo Chinese herbal which Containing 2% of Chinese herbal medicine.
With all ingredients of tested formula	13 (28.3)	Low doses of basic decoction.
With some of ingredients of tested formula	4 (8.7)	Placebo will constitute granules with 10% active core ingredients as below: *White Peony Root* 10 g *Processed Liquorice* 3 g *Immature Bitter Orange* 8 g *White Atractylodes Rhizome* 15 g.
With CHM ingredients plus other ingredients (e.g. excipients, coloring agents, flavoring agents)	4 (8.7)	We compromise the raw materials for the placebo including 10% of Chinese medicine, food color and artificial flavors.
Dosage	9 (19.6)	2% (*n* = 2): Containing 2% of *Qing’E* pills;
		5% (*n* = 4): Placebo made from 1/20 doses;
		10% (*n* = 3): 10% of the original dose.
Without CHM ingredients	29 (63.0)	Placebo composition of maltodextrin, lactose, edible pigment, taste masking agent.
Excipients	28 (60.8)	Flour, starch, dextrin, cornstarch, rice starch, Lactose, maltodextrin, wheat powder, medical carbon, etc.
Coloring agents	11 (23.9)	food color, Lemon yellow powder, brown powder, coloring materials, caramel, etc.
Flavoring agents	12 (26.1)	artificial flavors, citric acid, tea essence, taste masking agent, bitter taste agent, etc.
Other agents	5 (10.9)	polyethylene glycol 6000, denatonium benzoate, Silicon dioxide, Iron oxide black, Iron oxide red, etc.
Pharmacological tests	2 (4.3)	Placebo has no therapeutic effect.

## Discussion

This study identified 363 CHM interventional trials with placebo-controlled design that were registered from 2002 to 2017; this accounted for 40.8% of the WHO-registered CHM interventional trials. Although the usage of placebo steadily increased each year, reaching a maximum in 2017 with 97 CHM placebo-controlled trial registrations, this review found that some problems had existed in the following aspects of placebos, namely: (1) reporting information about the placebo (i.e. its physical characteristics); (2) ethics of placebo application; (3) placebo preparation and production; and (4) methods of placebo assessment; and (5) PICOS (participant, intervention, comparison, outcome and study type) related with placebo design.

### Reporting about the placebo

The placebo design is usually included in the reporting record of a CHM trial registration. Although the WHO Trial Registration Data Set (TRDS) requires the description of control intervention (e.g. placebo) should be appropriately detailed, this study found that the rates of reporting placebo-related information were very low. In descending order, these rates were: dosage form (84.6%), administration time (40.8%), physically identical test (14.3%), placebo composition (12.7%), placebo quality control (3.9%), pharmacologically inert testing (0.6%), and evaluation of successful blinding (0.6%). It’s no surprise that this same situation also appears in the publications of CHM placebo-controlled trials [22]. For example, physically identical and pharmacologically inert are the basic requirements for a placebo [23], but fewer available published CHM placebo-controlled trials have reported the placebo pharmacologically inert tests or physical similarity results [24, 25].

These findings highlight the need for establishing standard reporting items for placebo-related information. In 2017, a reporting guideline of Consolidated Standards of Reporting Trials (CONSORT) extension for CHM formulas was published, which included five reporting items for CHM-placebo information, namely 1) name and amount of each ingredient of the placebo; 2) description of the similarity of placebo with the intervention (e.g., color, smell, taste, appearance, packaging); 3) quality control and safety assessment, if any; 4) administration route, regimen, and dosage; and 5) production information: where, when, how, and by whom the placebo was produced [26].

For clinical research, registration is the first important step which adequately reflected the trial design, including the placebo design. Without detailed reporting of placebos, the purposes of any trial registration, including efficient, objective, accurate transfer of trial information and progress in health care, could be undermined [27]. Therefore, it is recommended that a complete description of the placebo in a trial registration record should be appropriately reported the above five items.

### Ethics of placebo application

Ethical issues must be considered prior to designing a placebo-controlled trial of CHM. First, the ethical use of placebos should include receiving approval from the ethics committee and obtaining informed written consent from all participants [28]. This study, however, found that except 251 (69.4%) trials had ethics approval,18 (5%) trials reported they did not obtain ethics approval, and 94 (25.9%) trials did not report whether they had such approval or not.

A second ethical consideration is the type of condition appropriate for placebo design and usage. There were many types of conditions involved in this study and the top three were diseases of circulatory system (12.4%), digestive system (9.9%) and infectious and parasitic diseases (9.6%), which included both of organic diseases and functional diseases. In comparison, previous studies have reported that the common types of diseases considered for placebo usage were functional disease and self-limiting disease, such as functional gastrointestinal disease (e.g. Irritable bowel syndrome) [29–31]. In addition, some scholars have suggested that the placebo design and application may be acceptable for the following three categories of conditions: self-healing disease (e.g. acute viral hepatitis); diseases without specific treatments (e.g. multiple viral infectious diseases); and chronic conditions with mild symptoms, where no adverse events are expected from delayed treatment (e.g. rheumatoid arthritis) [32].

Unfortunately, one of the weaknesses of CHM trial registration records is that inadequate details of study diseases were provided (e.g. early or late, mild or severe, etc.), making it difficult to examine the type of condition appropriate for the use of placebo. In that case, the ethics approval of a trial must be required to provide in the registration system.

### Placebo preparation and production

The core issue of placebo design is the preparation and production of a placebo, which included three critical elements: (1) selection of dosage form; (2) selection of materials and amount of each ingredient; and (3) quality control of placebo production. However, in practice, it is a challenge to design a perfectly matching placebo that makes blinding easy [33].

Our results found that except for 56 trials (15.4%) which did not report placebo dosage form, a total of 12 forms of dosage were identified. The most common one was granule (37.5%), followed by capsule (13.5%) and decoction (11.3%). Some scholars have pointed out that it is easier to design physically identical capsules than other dosage forms [34], but the capsule was not the first choice in this study. The dosage form of a placebo is the primary factor affecting the design of placebo ingredients and dosage. For example, for granule placebo, it is recommended to choose excipients that mimic the color and taste of the testing CHM, and then to spray them with CHM to create identical smell [35]. It is essential that these placebos, even though they have ingredients from the intervention, should be inert. For decoction placebo, the protocol is similar, that is, the placebo contains a low concentration (e.g. 5–10%) of the intervention CHM [36].

In terms of ingredients of the placebo, two main categories were discovered in this study: (1) with CHM ingredients; and (2) without CHM ingredients. For the first category, there were three common types: (a) all tested CHM formula ingredients were included in the placebo; (b) some of the tested CHM formula ingredients were included in the placebo; and (c) tested CHM formula ingredients plus other agents were included in the placebo. Normally, the dosages of applied CHM ingredients were in low percentage, such as 2, 5% or 10% only. For those without CHM ingredients, the placebo was mainly composed of excipients, such as flour, starch, dextrin, plus coloring agents and flavoring agents. In previous study, Tang XD et al. have suggested that it is better, in general, to choose unbiasedness foodstuffs as excipients for CHM placebos. If there are difficulties in simulating color, taste and smell of CHM, a low dosage of CHM ingredients could be added in the placebo, but the pharmacologically inertness meeting the acceptance of professional experts should be ensured [37].

It is important to make sure the quality of placebo production should be under rigor control. As with any CHM intervention, quality control of placebo production should follow a strict, systematic procedure, including requirements from selection of raw materials to production of the final products [38]. Unfortunately, this issue has largely been ignored. In this study, among 363 included trials, only 14 trials reported the manufacturer, e.g., Sanjiu Medical & Pharmaceutical Co., Ltd., Jiangyin Tianjiang Pharmaceutical Co., Ltd. Therefore, it is generally recommended that the manufacturer of placebo should have certification of Good Manufacturing Practice (GMP) [39].

### Methods of placebo assessment

Placebo assessment deserves special attention because ensuring that a placebo has two qualities, e.g., physically identical and pharmacologically inertness, is essential. Any activity due to the placebo will affect the relative efficacy of the intervention, whether positively or negatively [40]. According to this study, 52 trials described the similarity of placebo with the CHM intervention in terms of color, smell, taste, appearance, package, shape, size, weight, texture, etc., and 2 trials reported testing for pharmacological inertness. However, no trial had reported any planned testing methods or any objective indicators for placebo assessment. The selection of methods for placebo assessment can also affect the trial’s blinding evaluation.

Previous studies have reported two methods for placebo assessment: (1) artificial scoring (e.g., the placebo score from judgement of different assessors) [41]; and (2) bionic electronic evaluation (e.g., use of artificial intelligence products to evaluate the smell and taste of placebo) [42]. Given the subjectivity of artificial scoring, some scholars have developed a placebo quality checklist with an improved version for artificial scoring [43], with the goal of standardizing the artificial evaluation system. By contrast, using bionic electronic nose and electronic tongue to test the smell and taste of the placebo might be a feasible way to collect objective indicators of placebo evaluation [44, 45]. Therefore, using both artificial scoring and electronic evaluation, based on a predefined weight coefficient of each indicator, could help ensure relatively objective assessment of a placebo and its successful rate of blinding.

### PICOS (participant, intervention, control, outcome and study type) related with placebo design

A good design of placebo not only depends on the quality of placebo itself, such as placebo preparation, production and so on, it also related to the overall quality of its trial design, particular in the design of participant, intervention, comparison, outcome and study type (PICOS). This study found that the common reporting design of PICOS in the trial included (1) the circulatory system diseases; (2) granule of CHM formula; (3) solely placebo as control; (4) with objective and subjective outcomes; and (5) interventional clinical trial in phase II.

In a CHM placebo-controlled trial, the first consideration could be whether participants/diseases are suitable for use of placebo and under the ethical requirements. This issue has been discussed in the above (e.g., *Ethics of placebo application*). For the intervention, compared with a single herb or a chemical drug, a CHM formula has more special, more complex features in color, smell and taste, thus increasing the difficulty in placebo simulation. Thus, the placebo design of the CHM formula has been the topic of lively discussion for years. Some scholars have indicated that learning and accumulating experiences related to CHM-placebo creation are considered critical [46, 47]. For the control group, some scholars have believed that the selection of placebo control plus active drug control (e.g., a three-arm study) could be more suitable for clinical trials evaluating new drugs, especially drugs for diseases that are susceptible to psychological impact, such as analgesia, depression, dementia, etc. [48, 49]. Some other scholars have suggested that an add-on design, that is, conducting baseline treatment in both the experimental group and placebo group, could be used in organic diseases with effective treatment and aims to reduce mortality or morbidity of diseases [50]. Thus, an appropriate design of control group could be according to characteristics of the participants/diseases being studied. For the selection of outcomes, placebo effect also needed to be considered [51, 52]. For example, some scholars have recommend using subjective indicators to evaluate efficacy of the CHM intervention compared with the placebo comparator [53]. In this study, a total of 239 (65.8%) trials included the subjective outcomes. For study type, it is generally suggested that placebos are usually used in CHM interventional clinical study phases of II and III [54, 55]. This study, however, found that some trials were not within this scope (e.g., in the phase I or IV). Thus, the placebo design and application needed to be further standardized.

### Limitations

Our study has some limitations. First, this study included CHM trials with placebo control that were registered up to 31 December 2017. Any trials registered in regions which had not yet been included in ICTRP by that time have not been included. Second, this study mainly relied on registration information collected from specific registries, not on study protocols and publications. Third, some CHM placebo-controlled trials were conducted without being registered. This means that our results are not necessarily comprehensive. However, we believe that the general trends indicated by the analysis of the information we did use, even if incomplete, are valid.

### Recommendations

To improve the quality of placebo design in CHM clinical trials, we recommend as follows:

1. The criteria of whether to design or apply a placebo should be strictly followed the scientific and ethical requirements. Ethical approvals must be reported in the trial registration records, which is also required in the trial registration checklist of WHO trial registration data set (TRDS) (e.g., Item 21: Ethics review) [56].
2. Physical identical and pharmacological inert are the basic requirements for placebo design. Currently, it is difficult to makeup a perfectly matching placebo of CHM formula because of its special color, taste and smell. Thus, a standardized methodological procedure for designing CHM placebos should be developed.
3. As blinding assessment is very important to placebo-controlled trials, it is recommended to adopt or design the appropriate method(s) for placebo evaluation, such as the combination of artificial scoring and electronic evaluation.
4. Factors of PICOS, including the type of participants, CHM intervention and outcome, and the selection of control group design, should be seriously taken into consideration when designing a CHM placebo-controlled clinical trial.
5. Reporting of placebo characteristics information are encouraged as detailed as applicable, including composition, physical similarity, pharmacologically inert test, quality control, evaluation method(s) and so on.

## Conclusion

In summary, currently, the placebo design in WHO-registered CHM trials is not optimal particular in its preparation, application, evaluation and reporting, which undermining their intended value in such trials. The development of a full set of CHM-placebo standards, including usage requirements, preparation specifications, quality assessment and reporting guideline, could alleviate this problem. This could be achieved by combined efforts of health professionals, research scientists and pharmaceutical manufacturers.

## Supplementary information

**Additional file 1: S**_**1**_**.** Registration ID of the included trials. **S**_**2**_**.** Detailed information on physically identical testing of placebos reported in CHM trial registrations. **S**_**3.**_ Detailed information on manufacturers of placebos reported in CHM trial registrations.

## Data Availability

The original data used for this study can be freely downloaded from ICTRP search portal at http://apps.who.int/trialsearch/ and through hyperlinks to access the specific registries.

## Abbreviations

ANZCTR: Australian New Zealand Clinical Trials Registry
CFDA: China Food and Drug Administration
ChiCTR: Chinese Clinical Trial Registry
CHM: Chinese herbal medicines
CRiS: Clinical Research Information Service-Republic of Korea
CTRI: Clinical Trials Registry-India
DRKS: German Clinical Trials Register
EU-CTR: EU Clinical Trials Register
FDA: Food and Drug Administration
GMP: Good Manufacturing Practice
ICTRP: International Clinical Trials Registry Platform
IRCT: Iranian Registry of Clinical Trials
ISRCTN: International Standard Randomized Controlled Trial Number Register
JPRN: Japan Primary Registries Network
NTR: Netherlands National Trial Register
PACTR: Pan African Clinical Trial Registry
PICOS: Participant, Intervention, Control, Outcome and Study Type
RCT: Randomized controlled trial
ReBec: Brazilian Clinical Trials Registry
REPEC: Peruvian Clinical Trials Registry
RPCEC: Cuban Public Registry of Clinical Trials
SLCTR: Sri Lanka Clinical Trials Registry
TCM: Traditional Chinese medicine
TCTR: Thai Clinical Trials Register
TRDS: Trial Registration Data Set
WHO: World Health Organization
WM: Western Medicine
