# Supplementary material for: Placebo design in WHO-registered trials of Chinese herbal medicine need improvements
Source: BMC Complement Altern Med. 2019 Nov 6;19:299. doi: 10.1186/s12906-019-2722-2 (PMC6836479; doi:10.1186/s12906-019-2722-2)
Supplement: Supplementary file 1 — Additional file 1: S1. Registration ID of the included trials. S2. Detailed information on physically identical testing of placebos reported in CHM trial registrations. S3. Detailed information on manufacturers of placebos reported in CHM trial registrations. [file 12906_2019_2722_MOESM1_ESM.pdf]

S_1: Registration ID of the included trials.

ChiCTR (N=237)

ChiCTR-IOC-17013954	ChiCTR-IOC-17013974	ChiCTR-IOR-17013779	ChiCTR-IOR-17013699
ChiCTR-INR-17013653	ChiCTR-IOR-17013646	ChiCTR-IIR-17013609	ChiCTR-IOR-17013577
ChiCTR-IIR-17013532	ChiCTR-IOR-17013498	ChiCTR-IOR-17013491	ChiCTR-IOR-17013390
ChiCTR-IOR-17013378	ChiCTR-IIR-17013275	ChiCTR-INR-17013222	ChiCTR-IOR-17013189
ChiCTR-IOR-17013031	ChiCTR-INR-17013045	ChiCTR-IIR-17013018	ChiCTR-IPR-17012999
ChiCTR-IOR-17012681	ChiCTR-INR-17012674	ChiCTR-IPR-17012500	ChiCTR-IOR-17012471
ChiCTR-IOR-17012468	ChiCTR-IOR-17012421	ChiCTR-IOR-17012400	ChiCTR-IOR-17012337
ChiCTR-IIR-17012250	ChiCTR-IOR-17012238	ChiCTR-IPR-17012151	ChiCTR-INR-17012062
ChiCTR-IPR-17011990	ChiCTR-IPR-17011944	ChiCTR-INR-17011949	ChiCTR-IIR-17011940
ChiCTR-IIR-17011889	ChiCTR-IPR-17011876	ChiCTR-IOR-17011785	ChiCTR-IOR-17011746
ChiCTR-INR-17011569	ChiCTR-IOR-17011518	ChiCTR-INR-17011493	ChiCTR-IOR-17011429
ChiCTR-IPR-17011383	ChiCTR-INR-17011284	ChiCTR-IIR-17011106	ChiCTR-IPR-17011069
ChiCTR-IPR-17011035	ChiCTR-IOR-17011028	ChiCTR-IOR-17011036	ChiCTR-IPR-17010970
ChiCTR-INR-17010972	ChiCTR-INR-17010809	ChiCTR-IOR-17010765	ChiCTR-IOR-17010706
ChiCTR-INR-17010696	ChiCTR-INR-17010667	ChiCTR-IOR-17010600	ChiCTR-IOR-17010551
ChiCTR-INR-17010467	ChiCTR-IOR-17010397	ChiCTR-IOR-16007697	ChiCTR-OOC-16008573
ChiCTR-IIR-16009020	ChiCTR-INR-16007707	ChiCTR-INR-16009028	ChiCTR-INR-16009557
ChiCTR-IOR-16009312	ChiCTR-IOR-16010192	ChiCTR-IPR-16010154	ChiCTR-IOR-16010107
ChiCTR-IOR-16009992	ChiCTR-IOR-16009905	ChiCTR-IOR-16009880	ChiCTR-INR-16009756
ChiCTR-IOR-16009733	ChiCTR-INR-16009723	ChiCTR-INR-16009629	ChiCTR-IOR-16009637
ChiCTR-IOR-16009599	ChiCTR-IOR-16009469	ChiCTR-IOR-16009378	ChiCTR-IOR-16009331
ChiCTR-IOR-16009312	ChiCTR-IOR-16009148	ChiCTR-IOR-16009132	ChiCTR-IOR-14005387

ChiCTR-IOR-14005542	ChiCTR-IOR-15006876	ChiCTR-ICR-15006988	ChiCTR-IIR-15007492
ChiCTR-IOR-14005679	ChiCTR-IPR-14005429	ChiCTR-IOR-15006149	ChiCTR-IPR-14005665
ChiCTR-IPR-15006694	ChiCTR-IPR-15006945	ChiCTR-IOR-15006626	ChiCTR-IOR-15006768
ChiCTR-IOR-15006973	ChiCTR-IOR-15007526	ChiCTR-IOR-16007719	ChiCTR-IPR-14005357
ChiCTR-OPC-15006702	ChiCTR-IPR-15006020	ChiCTR-TRC-05000734	ChiCTR-TRC-09000565
ChiCTR-TRC-09000651	ChiCTR-TRC-10000770	ChiCTR-TRC-10000843	ChiCTR-TRC-10000957
ChiCTR-TRC-10000963	ChiCTR-TRC-10001004	ChiCTR-TRC-11001175	ChiCTR-TRC-11001255
ChiCTR-TRC-11001263	ChiCTR-TRC-11001375	ChiCTR-PRC-06000002	ChiCTR-TRC-11001409
ChiCTR-TRC-11001633	ChiCTR-TRC-08000204	ChiCTR-TRC-09000549	ChiCTR-TRC-10001518
ChiCTR-TRC-12002971	ChiCTR-TRC-11001274	ChiCTR-TRC-12003062	ChiCTR-TRC-12003174
ChiCTR-TRC-12003871	ChiCTR-TRC-11001755	ChiCTR-TRC-13003303	ChiCTR-TRC-13003324
ChiCTR-TRC-12001929	ChiCTR-TRC-13003326	ChiCTR-TRC-13003418	ChiCTR-TRC-13003431
ChiCTR-TRC-12002228	ChiCTR-TRC-13003531	ChiCTR-TRC-12002402	ChiCTR-TRC-13003552
ChiCTR-TRC-13003581	ChiCTR-TRC-13003605	ChiCTR-TRC-13003668	ChiCTR-TRC-13003742
ChiCTR-TRC-13003969	ChiCTR-TRC-13003233	ChiCTR-TRC-13003266	ChiCTR-TRC-13004029
ChiCTR-TRC-13003716	ChiCTR-TRC-14004180	ChiCTR-TRC-13003717	ChiCTR-TRC-13003780
ChiCTR-TRC-14004235	ChiCTR-TRC-14004403	ChiCTR-TRC-13003936	ChiCTR-TRC-14004613
ChiCTR-TRC-13004038	ChiCTR-TRC-13004072	ChiCTR-TRC-13004418	ChiCTR-TRC-14005098
ChiCTR-TRC-14005099	ChiCTR-TRC-14004215	ChiCTR-TRC-14004244	ChiCTR-TRC-14005185
ChiCTR-TRC-14004672	ChiCTR-TRC-14004758	ChiCTR-TRC-14005192	ChiCTR-TTRCC-14004406
ChiCTR-IOR-15005987	ChiCTR-IPR-15006194	ChiCTR-IPR-15007222	ChiCTR-TRC-05000622
ChiCTR-TRC-09000352	ChiCTR-TRC-09000668	ChiCTR-TRC-10000771	ChiCTR-TRC-10000836
ChiCTR-TRC-10001360	ChiCTR-TRC-11001712	ChiCTR-TRC-12002245	ChiCTR-TRC-12002330
ChiCTR-TRC-12002355	ChiCTR-TRC-12002447	ChiCTR-TRC-12002524	ChiCTR-TRC-12002539
ChiCTR-TRC-12002920	ChiCTR-TRC-12004849	ChiCTR-TRC-13003000	ChiCTR-TRC-13003323
ChiCTR-TRC-13003595	ChiCTR-TRC-13003985	ChiCTR-TRC-13004012	ChiCTR-TRC-13004045
ChiCTR-TRC-13004553	ChiCTR-TRC-14004156	ChiCTR-TRC-14004179	ChiCTR-TRC-14004515
ChiCTR-TRC-14004543	ChiCTR-TRC-14004699	ChiCTR-TRC-14004727	ChiCTR-TTRCC-13003732

ChiCTR-INR-15007378	ChiCTR-IOR-14005693	ChiCTR-IOR-14005750	ChiCTR-IOR-15007542
ChiCTR-IOR-15007615	ChiCTR-IOR-15007673	ChiCTR-IPC-15006295	ChiCTR-IPR-15006578
ChiCTR-TRC-07000037	ChiCTR-TRC-09000311	ChiCTR-TRC-09000425	ChiCTR-TRC-09000533
ChiCTR-TRC-10000845	ChiCTR-TRC-10001082	ChiCTR-TRC-10001105	ChiCTR-TRC-11001193
ChiCTR-TRC-11001220	ChiCTR-TRC-12001923	ChiCTR-TRC-12002054	ChiCTR-TRC-12002604
ChiCTR-TRC-12002850	ChiCTR-TRC-12002973	ChiCTR-TRC-12004548	ChiCTR-TRC-13003025
ChiCTR-TRC-13003037	ChiCTR-TRC-13003200	ChiCTR-TRC-13003202	ChiCTR-TRC-13003250
ChiCTR-TRC-13003321	ChiCTR-TRC-13003337	ChiCTR-TRC-13003683	ChiCTR-TRC-13003702
ChiCTR-TRC-13003738	ChiCTR-TRC-13004026	ChiCTR-TRC-14004529	ChiCTR-TRC-14004620
ChiCTR-TRC-14004765			

ClinicalTrials.gov (N=91)

NCT03372694	NCT03374111	NCT03320538	NCT03299322
NCT03290313	NCT03244605	NCT03228134	NCT03186625
NCT03185923	NCT03186079	NCT03173027	NCT03173040
NCT03147443	NCT03245710	NCT03072225	NCT03025152
NCT03027375	NCT03019042	NCT03025399	NCT03010241
NCT02795390	NCT03008798	NCT03135821	NCT02955134
NCT02945982	NCT02937207	NCT02929693	NCT02892357
NCT00393510	NCT00548223	NCT00554723	NCT00710164
NCT00887172	NCT00974519	NCT01104428	NCT01431352
NCT01441752	NCT00543426	NCT00602160	NCT01682551
NCT01745328	NCT01780181	NCT01805765	NCT00963287
NCT00974454	NCT01142479	NCT02319993	NCT01918722
NCT01965418	NCT01978730	NCT02232945	NCT02538692
NCT02590367	NCT02676713	NCT02490813	NCT02605655
NCT00676975	NCT00741936	NCT00799734	NCT00933725

NCT01045720	NCT01138930	NCT01271647	NCT01274936
NCT01373476	NCT01389362	NCT01452477	NCT01715740
NCT01879514	NCT01939236	NCT02027194	NCT02135250
NCT02153840	NCT02633878	NCT02641886	NCT00153751
NCT00904592	NCT01116167	NCT01223430	NCT01486186
NCT01502943	NCT01555320	NCT01580358	NCT01613183
NCT01695850	NCT01780480	NCT01840761	NCT01844050
NCT01913418	NCT01926834	NCT02313610	

ANZCTR (N=17)

ACTRN12617001247369	ACTRN12607000380493	ACTRN12608000133336	ACTRN12607000255482
ACTRN12608000619347	ACTRN12612000209897	ACTRN12612001181897	ACTRN12614000493640
ACTRN12612000128897	ACTRN12613000382774	ACTRN12608000132347	ACTRN12608000468325
ACTRN12608000618358	ACTRN12608000630314	ACTRN12609000558224	ACTRN12613000229774
ACTRN12614001172695			

ISRCTN (N=10)

ISRCTN70292892	ISRCTN23374750	ISRCTN69696725	ISRCTN34440093
ISRCTN19413357	ISRCTN66631530	ISRCTN90063632	ISRCTN06129599
ISRCTN12702489	ISRCTN70448213		

JPRN (N=3)

JPRN-UMIN000003954	JPRN-UMIN000006881	JPRN-UMIN000004401	

CRiS (N=3)

KCT0002387	KCT0002374	KCT0001348	

IRCT (N=2)

IRCT2017052712438N23	IRCT2017060434328N1		

S_2: Detailed information on physically identical testing of placebos reported in CHM trial registrations

Table 1. Description of the physical similarity of placebo with the CHM intervention

Identical items [1]	Report, N=52 (%)
Color, taste, and smell	18 (34.6)
**Color**	**27 (51.9)**
**Smell**	**27 (51.9)**
**Taste**	**25 (48.1)**
**Appearance**	**24 (46.2)**
Package	14 (26.9)
Shape	9 (17.3)
Size	7 (13.5)
Weight	6 (11.5)
Texture	4 (7.7)
Frequency and duration	2 (3.8)
Dosage form	2 (3.8)
Administration route	1 (1.9)

[1] In the contents of physical identical testing of the placebo, there included 13 aspects presented in the above Table 1. All trials (n=52) mentioned more than one aspect (e.g., *The smell, color and apparent package of placebo are as close as possible to PTQX.*), which were counted in different columns, respectively. Thus, the total number was above 52. The reporting rates of four aspects of color, smell, taste and appearance were higher than 45%, which presented in the main manuscript (Table 2).

S$_3$: Detailed information on manufacturers of placebos reported in CHM trial registrations

Table 1. Information on placebo manufacturers of included CHM trial registrations [1]

Name	N=14 (%)	GMP Certification	City, Country	Website
**Dong-E E-Jiao Co., Ltd**	1 (7.1)	Yes	Shandong, China	http://www.dongeejiao.com/
**Sanjiu Medical & Pharmaceutical Co., Ltd**	2 (14.3)	Yes	Shenzhen, China	https://www.999.com.cn
**Beijing Tcmages Pharmaceutical Co., Ltd**	1 (7.1)	Yes	Beijing, China	http://sunwaycn.globalimporter.net/
**Sigma Pharmaceuticals Pty Ltd**	1 (7.1)	Yes	Melbourne, Australia	https://sigmahealthcare.com.au/
**Boehringer Ingelheim Group company**	1 (7.1)	Yes	Ingelheim am Rhein, Germany	https://www.boehringer-ingelheim.com/
**Jiangyin Tianjiang Pharmaceutical Co., Ltd**	2 (14.3)	Yes	Jiangyin, China	http://www.tianjiang.com/
**SUN TEN Pharmaceutical Co., Ltd**	1 (7.1)	Yes	New Taipei, Taiwan	http://www.suntenglobal.com
**JZJT**	1 (7.1)	Yes	Nanchang, China	http://www.jzjt.com/
**No specific name**	4 (28.6)	Yes	-	-

[1] Among 14 trials that provided related information on placebo manufacturers, 10 trials reported the specific name of placebo manufacturers (n=8), and 4 trials only reported the manufacturers (e.g. No specific name) with GMP certification. Thus, more details (e.g. location, website and whether have GMP or not) of the 8 manufacturers were added through website search according to their name. No specific information were represented by "-".
